# Starch Origin and Thermal Processing Affect Starch Digestion in a Minipig Model of Pancreatic Exocrine Insufficiency

**DOI:** 10.1155/2015/872872

**Published:** 2015-05-12

**Authors:** Anne Mößeler, Sandra Vagt, Martin Beyerbach, Josef Kamphues

**Affiliations:** ^1^Institute for Animal Nutrition, University of Veterinary Medicine Hannover, Foundation, Bischofsholer Damm 15, 30173 Hannover, Germany; ^2^Department for Biometry, Epidemiology, and Information Processing, University of Veterinary Medicine Hannover, Foundation, Bünteweg 2, 30559 Hannover, Germany

## Abstract

Although steatorrhea is the most obvious symptom of pancreatic exocrine insufficiency (PEI), enzymatic digestion of protein and starch is also impaired. Low praecaecal digestibility of starch causes a forced microbial fermentation accounting for energy losses and meteorism. To optimise dietetic measures, knowledge of praecaecal digestibility of starch is needed but such information from PEI patients is rare. Minipigs fitted with an ileocaecal fistula with (*n* = 3) or without (*n* = 3) pancreatic duct ligation (PL) were used to estimate the rate of praecaecal disappearance (pcD) of starch. Different botanical sources of starch (rice, amaranth, potato, and pea) were fed either raw or cooked. In the controls (C), there was an almost complete pcD (>92%) except for potato starch (61.5%) which was significantly lower. In PL pcD of raw starch was significantly lower for all sources of starch except for amaranth (87.9%). Thermal processing increased pcD in PL, reaching values of C for starch from rice, potato, and pea. This study clearly underlines the need for precise specification of starch used for patients with specific dietetic needs like PEI. Data should be generated in suitable animal models or patients as tests in healthy individuals would not have given similar conclusions.

## 1. Introduction

The pancreatic duct ligated (PL) pig or minipig is an established model for studying pancreatic exocrine insufficiency (PEI) in humans and for obtaining information about effects of PEI on digestive processes and for optimising enzyme substitution therapy [1–5].

Coefficients of absorption for fat and protein over the total tract are much more decreased in the case of PEI than that of starch. Overall, the topic of carbohydrate malabsorption in the case of PEI has still to be quantified accurately [6, 7]. Amylorrhea is not a typical symptom of patients with PEI as loss of pancreatic amylase causes only a mild malabsorption of carbohydrates [8, 9] due to extrapancreatic amylases and the very high activity of the microflora resulting in an almost complete compensative starch fermentation in the hindgut [10]. In PL-pigs with complete loss of exocrinal pancreatic function and without pancreatic enzyme substitution therapy, starch digestibility over the entire alimentary tract did not differ from values observed in healthy controls [10, 11]. In human PEI patients, Ladas et al. [7] found a malabsorption rate of about 10 to 30% for complex carbohydrates estimated over the entire gastrointestinal tract, indicating that the capacity for carbohydrate fermentation in the human hindgut is less pronounced than in pigs. Analyses of stool samples are a feasible method for direct quantification of net carbohydrate malabsorption in human patients but have the disadvantage of not allowing any differentiation between enzymatically and fermentative digestion of starch. Indirect parameters like hydrogen exhalation [12] enable estimation of the extent of bacterial fermentation in digestive processes of carbohydrates. In experimental studies, the use of fistulated animals enables the sampling of the ileal chyme, an interesting method to differentiate between digestive processes in the small intestine (mainly enzymatically digestion) and large intestine (exclusively fermentative digestion).

On the one hand, the fermentation of carbohydrates contributes to the patients' energy supply by providing short chain fatty acids [13, 14], but on the other hand there is also an increased gas production concomitantly [15], impairing the wellbeing of patients due to excessive intestinal formation of gas resulting in abdominal pain and flatulence [14, 16]. Furthermore, there is also a higher risk of fermentative diarrhoea [17] due to the excessive influx of carbohydrates into the hindgut.

As observed in a former study, the botanical origin of the starch had a marked effect on praecaecal digestibility in PL-pigs [23], but in that study only raw starch sources were used which does not mimic the situation in human nutrition. Therefore, this study aimed to test potential effects of the botanical origin as well as thermal treatment (cooking) of the starch on praecaecal digestibility in minipigs with or without experimentally induced PEI.

The results of this study should contribute to optimising dietary recommendations for human patients suffering from PEI.

## 2. Material and Methods

All efforts were made to minimise both the stress for the individual animal and the numbers of animals used. The procedures used in this study were conducted in accordance with the German Animal Welfare Act and with the European Council Directive of 24 November 1986 (86/609/EEC) and were approved by the Ethics Committee on Animal Welfare of the Hannover District Government.

### 2.1. Animals

Altogether, 6 adult female minipigs, fitted with an ileocaecal reentrant fistula (according to the method described by Tabeling et al. [11]), were used for this study. In PL-pigs (*n* = 3), additionally, a ligation of the* ductus pancreaticus accessorius* was performed to induce the PEI experimentally. In all pigs activity of faecal chymotrypsin was measured (test kit purchased from Immundiagnostik AG, Wiesenstrasse 4, 64625 Bensheim, Germany, catalogue number K6990) and only minipigs with a chymotrypsin activity < 0.900 U/g faeces were defined and used as PL-pigs. The other minipigs (*n* = 3) with an intact pancreas served as controls (C). The body weight of the minipigs used in the present study was 37.0 ± 2.50 kg (C) and 37.7 ± 5.74 kg (PL).

### 2.2. Test Design

Starch of different botanical origin was chosen to include starch sources of different granula size in this study (Table 1).

The different starch sources were tested in a screening-test established by Becker [24]. Therefore, the terms “digestibility rate” or “coefficient of starch absorption” were replaced by rate of “praecaecal Disappearance” (pcD). The test diet (including the starch to be tested and Cr_2_O_3_ as a marker) was fed only once without any adaptation period and chyme collection was performed over 8 h after the first occurrence of the green colour of the chyme indicated the arrival of the test diet at the fistula at the terminal ileum. In PL-pigs enzyme replacement therapy with Creon was stopped at least three days before the trial started and no enzymes were given during the test. In the evening before the test started the pigs were fed only 400 mL of a liquid diet (ProvideXtra DRINK by Fresenius Kabi Deutschland GmbH, Bad Homburg, Germany) to optimise gastric emptying and to avoid a carryover of nutrients of the diet from the previous evening into the test. A wash-out period of at least 48 h between two screening-tests was used to ensure a washout of Cr_2_O_3_ out of the praecaecal gastrointestinal tract. The different sources of starch were tested in a randomised order with every pig receiving all diets. For reasons of low palatability and incomplete feed intake one pig had to be replaced by another for the test diet containing raw amaranth.

### 2.3. Test Diet

The test meal consisted of 150 g of the starch to be tested, 25 mL of olive oil (Fa. Roth, Karlsruhe, Germany), 30 g of methylcellulose (Methocel, Sigma Aldrich Chemie, Germany), and 0.625 g Cr_2_O_3_ (Sigma Aldrich Chemie, 98% ≤ 50 *μ*m). Isolated starch and test diets were analysed using standard methods according to Naumann and Bassler [25]. Starch content was measured polarimetrically while amylose content of the starch was measured enzymatically using a test kit (K-AMYL, Megazyme, Bray Co., Wicklow, Ireland).

Chemical composition of the test diets is described in Table 2. As no isolated amaranth starch was commercially available a whole-grain meal was used for this trial. Therefore, this test meal contained a lower starch content as the total amount of diet was identical (see Table 2). To test whether the lower starch content of amaranth affected values of pcD, in addition, a higher amount of the raw amaranth whole-grain meal (212 g instead of 150 g) was fed to reach a starch uptake comparable to the other diets.

Thermal treatment of starch was performed by cooking over 20 minutes. The amount of water was adapted to allow continuous stirring of the starch mash with amounts of water differing from 2.5 (amaranth) up to 5 litres (potato) per kg starch. After cooking, starch was dried by freeze drying (freeze dryer: alpha 1–4 LSC, Martin Christ, Gefriertrocknungsanlagen GmbH, Osterode am Harz, Germany).

### 2.4. Sampling

Ileal chyme was collected by opening the reentrant fistula, closing the caecal fistula with a cover containing a membrane, and fixing a flexible bin to the ileal fistula. These bins were emptied frequently and replaced by new ones. Water and electrolytes were substituted (1 mL per gramme chyme collected) into the caecal fistula every two hours according to the method described by Tabeling et al. [11].

### 2.5. Sample Preparation and Analyses

Samples of ileal chyme were weighed and frozen immediately after collection, freeze-dried (freeze dryer: alpha 1–4 LSC, Martin Christ, Gefriertrocknungsanlagen GmbH, Osterode am Harz, Germany), and analysed regarding dry matter and starch (polarimetrically) according to Naumann and Bassler [25]. Cr_2_O_3_ was determined according to the method of Petry and Rapp [26]. The pcD was calculated according to Becker [24] and Mößeler et al. [23].

Statistical analysis was performed using SAS version 9.31 (SAS Institute Inc., Cary, NC, USA). For testing the effects of botanical origin and thermal processing of the starch within one group of animals a paired* t*-test was used while for the comparison of the results of the control and PL-pigs an unpaired* t*-test was used. Significant effects of the group were stated when *P* < 0.05.

## 3. Results

### 3.1. Effect of PEI on Praecaecal Disappearance Rate of Raw Starch

**Figure 1:**
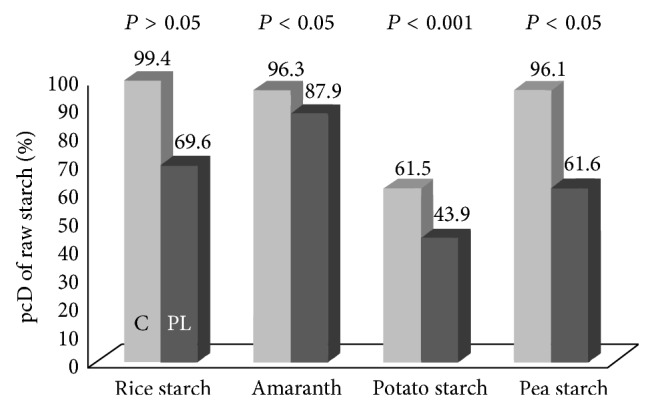
pcD of different sources of starch fed raw to control-pigs (C, light grey bars) and pancreatic duct ligated pigs (PL, grey bars).

The pcD of starch was significantly reduced in the PL-pigs compared to the controls for amaranth, potato starch, and pea starch. When raw rice starch was fed, there was also a large numerical difference regarding pcD between the controls and PL-pigs but due to high individual variation in the PL-pigs (69.6 ± 23.7) this difference reached no significance (see Figure 1).

### 3.2. Effect of PEI on Praecaecal Disappearance Rate of Thermal Treated Starch

**Figure 2:**
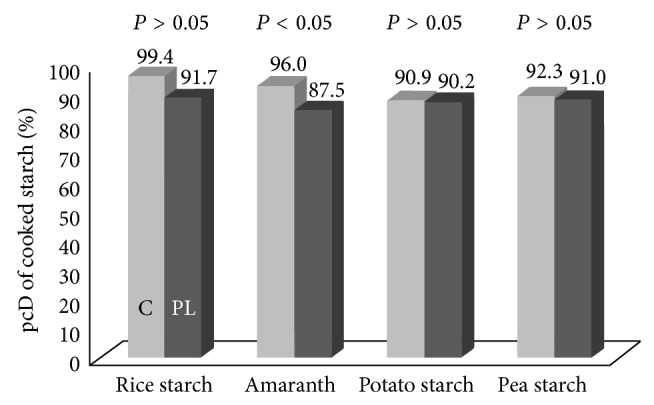
pcD of different sources of starch fed after thermal treatment to control-pigs (C, light grey bars) and pancreatic duct ligated pigs (PL, grey bars).

The pcD of starch was significantly reduced in PL-pigs compared to controls for cooked amaranth, while there was no significant effect of PEI when rice starch, potato starch, and pea starch were fed (see Figure 2).

### 3.3. Effect of Botanical Origin of Starch

In C-pigs the pcD of raw starch was high in general (>96%) but was significantly lower when raw potato starch was fed (61.5%), while there was no significant effect of botanical origin when the different sources of starch were fed cooked, with values varying from 91 to 99% (see Table 3).

In PL-pigs feeding of the raw potato starch resulted in the lowest pcD with values (43.9 ± 2.71%) being significantly lower (*P* < 0.005) than pcD observed after feeding amaranth (87.9 ± 3.50). When cooked starch was fed, the values differed between 87.5% (amaranth) and 91.7% (rice starch).

### 3.4. Effect of Thermal Processing

In both groups, cooking of starch resulted in a significant increase (*P* < 0.05) of pcD of potato starch. While thermal treatment did not affect pcD of starch in controls (except for potato starch), in the group PL-pigs, cooking of starch resulted in a significant increase in pcD for all sources of starch tested except for amaranth. It is worth mentioning that variation regarding pcD was very high in the PL-pigs for raw rice starch and raw pea starch. When these sources of starch were fed cooked the variation was much lower (see Table 3).

To check whether the high pcD of the starch from raw amaranth resulted from the lower starch content, additionally, a test meal with a higher amount of raw amaranth was fed to equalise the amount of starch per test meal. Neither in the C-pigs (97.5 ± 0.855) nor in the PL-pigs (81.3 ± 5.12) did the increased amount of the raw amaranth per meal (212 g/meal) have any effect on the pcD compared to the values observed after feeding 150 g of amaranth per meal.

## 4. Discussion

As determining praecaecal digestibility is of greatest relevance to evaluate the nutritive value of starch [27], use of fistulated animals is an established way to investigate questions dealing with this topic. There are many studies dealing with the effects of different types of cereals and further processing on digestibility of starch or glycemic index in healthy individuals [28, 29]. The term resistant starch is used to describe the starch escaping from enzymatic digestion in the small intestine [30].

The part of starch being digested and absorbed in praecaecal parts of the GIT is of special interest as postileal degradation of resistant starch is only performed by intestinal microflora, having beneficial effects on colonic health by degradation to butyric acid [31] but also resulting in an increased gas production (causing energy losses and meteorism increasing the risk for abdominal pain). Therefore, a high praecaecal digestibility of starch is intended in general, if no probiotic effects of resistant starch or a low glycemic index [28] are the focus of interest. In patients with PEI the lack of pancreatic amylases can be compensated to some extent by salivary amylases and digestive enzymes of the brush border membrane [32]. Nonetheless, it has to be considered that, especially in case of small intestinal bacterial overgrowth [33, 34], starch is also partly digested by microbial degradation in the small intestine resulting in increased intestinal gas production which can be used for diagnostic tests such as the hydrogen exhalation test [12].

The digestibility of starch is influenced by botanical origin [35] with content of amylose being one factor [29, 35]. The higher pcD of starch with low amylose content was confirmed in both groups of animals, but in both groups the pcD of raw pea starch was higher than for potato starch, although the amylose content in the pea starch was highest.

**Figure 3:**
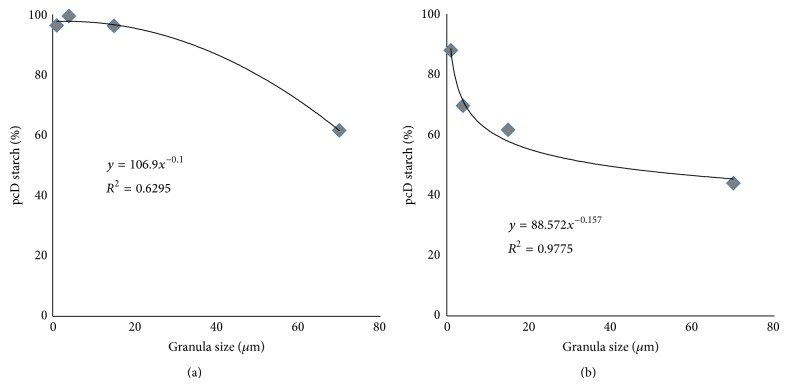
pcD of raw starch (%) in control-pigs (a) and PL-pigs (b) plotted against the granula size of the starch used (data taken from literature).

Furthermore, the digestibility was affected by granula size of the starch. The pcD of starch was lower for starch sources with larger granula size as a result of lower surface area [21]. Although the strength of this finding is limited due to the low number of starch sources used in this study this finding seems noteworthy and further sources of starch should be tested to verify this hypothesis. The very high pcD of amaranth starch is assumed to result from the very small granula size and the low amylose content of this starch. The correlation between granula size (taken from literature) (see Table 1) and pcD of different sources of starch is slight (smallest particle size resulting in highest pcD). It can be assumed that the higher surface area allows a better degradation and respectively digestion by extrapancreatic amylases; furthermore porosity of starch is another relevant aspect that needs to be taken into account as it might affect access of enzymes to the granule interior [36]. Interestingly in C-pigs three of four sources of starch were digested almost completely with no effect of granula size while the data of PL-pigs were best visualised by a logarithmic trend line (see Figure 3). As the surface area of a sphere is 4*πr* ^2^ but the volume is 4/3*πr* ^3^ the right hand curve is consistent with a role for particle size determining the surface area for enzymatic attack.

Cooking starch has been well known for a very long time and is a traditional method to increase praecaecal digestibility of starch and to reduce negative side effects of fermentation. It is well accepted that necessity of thermal processing is dependent on the source of starch; for example, raw potato starch (high proportion of resistant starch) is not adequate for human nutrition while most grains (breakfast cereals) can be used as food without thermal processing in healthy individuals.

The results of this study are of special interest for dietary treatment of human patients with PEI. Adult healthy minipigs are able to digest starch of different origin quite efficiently praecaecally; even the starch was not heat treated. Only raw potato starch was digested to a lower extent (*P* < 0.05) in C-pigs. This finding tallies well with that of Rerat [37] and is presumably due to the high content of resistant starch in potatoes [38]. In PL-pigs pcD of raw starch was distinctly reduced in comparison to C-pigs for most sources of starch. In patients with PEI raw sources of starch with a low pcD should be avoided to minimise the bacterial fermentation processes in the hindgut. Although treatment of PEI mainly focuses on lipase substitution, the widespread use of multienzyme products provides amylases as well as improving praecaecal starch digestibility. However, in case of use of monoenzyme products (isolated lipase) or when a diet low in fat is consumed (and therefore enzyme dosage is low when enzymes are dosed according to the amount of ingested fat [39]) there might be a lack of amylolytic enzymes. The optimisation of the praecaecal digestibility of starch is very important as resistant starch not being digested praecaecally is fermented almost completely in the hindgut and might result in massive intraintestinal gas production. In* in vitro* tests fermentation of 1 g of starch in ileal chyme of PL-pigs resulted in a production of up to 160 mL of gas [15]. Although the consumed starch is quite often cooked or processed thermally in human nutrition [40], there is food (e.g., breakfast cereals) that is not processed. Therefore, the results observed here are of interest for patients with PEI. Particularly as there is a trend for raw vegetarian food in some parts of the population as “health food” this might be relevant. Using raw foodstuffs containing starch in the nutrition of PEI patients should be done carefully and on an individual basis of tolerance.

The screening-test model used in this study seems to be an interesting and suitable method not only for testing pancreatic enzyme preparations* in vivo* [41] but also for screening different foodstuffs.

This study clearly underlines the need for botanic characterisation of starch in nutrition and dietetics. The fact that ranking of starch pcD differed between C- and PL-pigs emphasises the need for investigations on specific animal models or patients when trying to optimise diet formulation for special dietetic purposes.

## Figures and Tables

**Table 1:** Granula size and amylose content (%) of starch sources used in this study.

		Rice starch	Amaranth meal	Potato starch	Pea starch
Granula size (*μ*m)^∗^		2–10	0.5–2.0	15–100	10–45

Granula size (*μ*m)^∗∗^		4	1	70	15

Amylose content (%)	Raw	6.3	1.53	8.68	20.5
	Cooked	11.1	1.86	18.3	27.0

^∗^According to literature [18–22].

^∗∗^Values used for calculating correlation between starch granula size and pcD.

**Table 2:** Nutrient concentration (g/kg DM) of the test diets containing different sources of starch (analysed values).

	Crude ash	Crude protein	Crude fat	Starch	Sugar
Rice starch					
Raw	7.73	3.29	140	672	<1
Cooked	9.95	3.53	133	690	1.66
Amaranth meal					
Raw	23.4	107	190	431	11.7
Cooked	25.6	110	187	476	11.3
Potato starch					
Raw	7.52	2.25	131	605	<1
Cooked	7.37	2.61	126	618	1.66
Pea starch					
Raw	7.69	3.40	126	683	1.20
Cooked	8.15	2.32	134	605	2.83

**Table 3:** PcD of starch (%) in control- and PL-pigs when starch of different botanical origin was used either raw or cooked.

	Control-pigs		PL-pigs	
	Raw	Cooked	Raw	Cooked
Rice starch	99.4 ± 0.372^∗a^	99.4 ± 0.559^a∗^	69.6 ± 23.7^∗ab^	91.7 ± 7.01^#ab^
Amaranth	96.3 ± 3.07^∗a^	96.0 ± 2.19^∗a^	87.9 ± 3.50^∗a^	87.5 ± 3.08^∗a^
Potato starch	61.5 ± 1.90^∗b^	90.9 ± 9.61^#a^	43.9 ± 2.71^∗b^	90.2 ± 4.82^#ab^
Pea starch	96.1 ± 3.30^∗a^	92.3 ± 4.65^∗a^	61.6 ± 20.6^∗ab^	91.0 ± 2.37^#b^

Different symbols mark significant effects of thermal treatment within one group of animals when starch of one botanical source was fed (*P* < 0.05).

Different letters mark significant effects of botanical origin within one group of animals when starch was fed either raw or cooked (within one row) (*P* < 0.05).
